# P-597. Gender, Geography, and Dengue Deaths: Unraveling the High Mortality Crisis in South-East Asia and Bangladesh

**DOI:** 10.1093/ofid/ofaf695.811

**Published:** 2026-01-11

**Authors:** Supta Sarker, Md Shariful Amin Sumon

**Affiliations:** icddr,b, Dhaka, Dhaka, Bangladesh; icddr,b, Dhaka, Dhaka, Bangladesh

## Abstract

**Background:**

Dengue continues to pose a significant public health risk, with South-East Asia seeing the highest fatality rates worldwide. During 2023–2024, Bangladesh had an unprecedented increase in cases and deaths, characterized by significant gender differences. This study consolidates geographical patterns and factors contributing to the disproportionate effect of dengue, emphasizing the heightened case fatality rate (CFR) in South-East Asia and the crisis of female death in Bangladesh.

**Figure:**
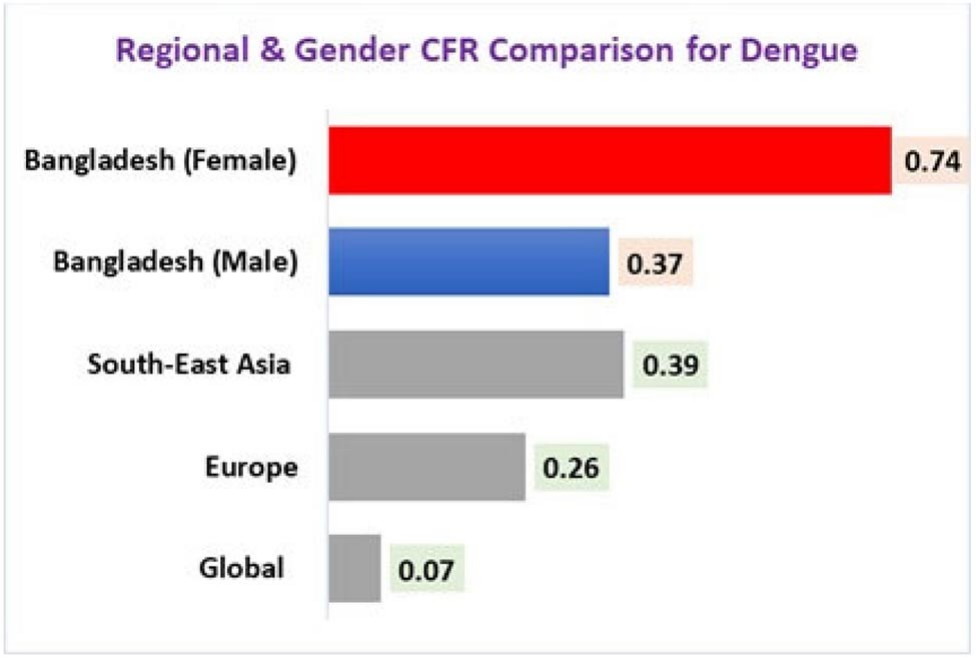
Regional and Gender CFR in Dengue Comparison

**Methods:**

Retrospective data from 2017 to 2024 were obtained from WHO surveillance, ADVA, DGHS reports, and hospital records of NS1/IgM-confirmed patients. Qualitative insights were obtained from 24 healthcare practitioners in Bangladesh. Serotyping data (N=511) from IEDCR/NILMRC were examined.

**Results:**

The case fatality rate (CFR) in South-East Asia stands at 0.39%, significantly exceeding the global average of 0.07%. Notably, Bangladesh recorded a CFR of 0.52% in 2023, the highest in the world. Females exhibited a mortality risk twice that of males (CFR 0.74% compared to 0.37%), with a particular emphasis on low-income women aged 31–40. Primary factors identified were delayed care (70.8%), climate shifts (66.7%), healthcare gaps (62.5%), urban density (58.3%), and serotype dominance (DENV-2: 68.1%). Dhaka's case fatality rate (0.87%) surpassed that of rural areas, despite a lower number of cases.

**Conclusion:**

The interplay of biological, socioeconomic, and systemic failures intensifies dengue mortality in South-East Asia, with Bangladesh serving as a prime example of gender-geographic inequities. Immediate and focused interventions, including timely access to care, vector control measures, and gender-sensitive policies, are essential to address this crisis.

**Disclosures:**

All Authors: No reported disclosures

